# Establishing Ghanaian adult reference intervals for hematological parameters controlling for latent anemia and inflammation

**DOI:** 10.1111/ijlh.13296

**Published:** 2020-09-03

**Authors:** Abigail S. A. Bawua, Kiyoshi Ichihara, Rosemary Keatley, John Arko‐Mensah, Yvonne Dei‐Adomakoh, Patrick F. Ayeh‐Kumi, Rajiv Erasmus, Julius Fobil

**Affiliations:** ^1^ Department of Biological Environmental & Occupational Health Sciences University of Ghana School of Public Health Legon Ghana; ^2^ Department of Clinical Laboratory Sciences Faculty of Health Sciences Yamaguchi University Graduate School of Medicine Ube Japan; ^3^ Medlab Ghana Ltd. (A Member of Synlab) Accra Ghana; ^4^ Department of Hematology University of Ghana Medical School College of Health Sciences Korle‐Bu University of Ghana Legon Ghana; ^5^ Department of Microbiology University of Ghana Medical School College of Health Sciences Korle‐Bu University of Ghana Legon Ghana; ^6^ Division of Chemical Pathology University of Stellenbosch Cape Town South Africa

**Keywords:** complete blood counts, iron markers, latent abnormal values exclusion method, latent anemia, multiple regression analysis, parametric method, SD‐ratio

## Abstract

**Background:**

In Ghana, diagnostic laboratories rely on reference intervals (RIs) provided by manufacturers of laboratory analyzers which may not be appropriate. This study aimed to establish RIs for hematological parameters in adult Ghanaian population.

**Methods:**

This cross‐sectional study recruited 501 apparently healthy adults from two major urban areas in Ghana based on the protocol by IFCC Committee for Reference Intervals and Decision Limits. Whole blood was tested for complete blood count (CBC) by Sysmex XN‐1000 analyzer, sera were tested for iron and ferritin by Beckman‐Coulter/AU480, for transferrin, vitamin‐B12, and folate was measured by Centaur‐XP/Siemen. Partitioning of reference values by sex and age was guided by “effect size” of between‐subgroup differences defined as standard deviation ratio (SDR) based on ANOVA. RIs were derived using parametric method with application of latent abnormal values exclusion method (LAVE), a multifaceted method of detecting subjects with abnormal results in related parameters.

**Results:**

Using SDR ≥ 0.4 as a threshold, RIs were partitioned by sex for platelet, erythrocyte parameters except mean corpuscular constants, and iron markers. Application of LAVE had prominent effect on RIs for majority of erythrocyte and iron parameters. Global comparison of Ghanaian RIs revealed lower‐side shift of RIs for leukocyte and neutrophil counts, female hemoglobin and male platelet count, especially compared to non‐African countries.

**Conclusion:**

The LAVE effect on many hematological RIs indicates the need for deliberate secondary exclusion for proper derivation of RIs. Obvious differences in Ghanaian RIs compared to other countries underscore the importance of country‐specific RIs for improved clinical decision‐making.

## 1 INTRODUCTION

Laboratory tests serve many purposes, including disease diagnosis and monitoring, predicting risk, and managing therapy. The critical contribution of laboratory tests in healthcare delivery is largely dependent on availability of appropriate information supporting proper interpretation.^1^, ^2^ Basically, this information is provided in the form of a reference interval (RI)^2^, ^3^, ^4^ or the central 95% range of the distribution of reference values (RVs) from well‐defined healthy individuals.^2^, ^3^

Considering the relevance of RIs, the International Federation of Clinical Chemistry (IFCC) has recommended that each laboratory establishes its own reference values and estimates the corresponding RIs using well‐defined procedures. Based on the IFCC's recommendation, the Clinical and Laboratory Standard Institute (CLSI) published the C28‐A3 guideline in determining RI for quantitative clinical laboratory tests.^5^

Following the guideline, several RI studies involving hematological parameters have been conducted in recent years in African countries.^6^, ^7^, ^8^, ^9^, ^10^ However, these reported RIs were discordant.^11^ This is probably attributable to either small sample size, loose criteria for volunteer recruitment, not accounting for latent diseases, or use of nonparametric method for analysis that is susceptible to extreme values.^12^, ^13^, ^14^ For improved derivation of RIs, country‐based multicenter study was recommended in 2012 by the IFCC Committee on Reference Intervals and Decision Limits (C‐RIDL).^15^ A harmonized protocol was issued by C‐RIDL, which optimized the methods for recruitment, sampling, measurement, secondary exclusion, and statistical analyses to allow for reproducible and standardized derivation of RIs.^12^, ^15^ Studies following the C‐RIDL protocol were recently conducted in Turkey^16^ and Kenya^11^ which established RIs from well‐defined healthy volunteers.

In Ghana, a few studies have been conducted to establish hematological RIs,^13^, ^17^, ^18^, ^19^ but none employed a prescribed well‐designed protocols as in other African studies, and these RIs may not be suitably applied to general use. As a matter of fact, most clinical laboratories continue to depend on hematological RIs provided by manufacturers. Therefore, it is crucial to establish RIs specific to the Ghanaian population by applying improved methodologies, and to serve as the RIs for unbiased clinical decision‐making. As a part of the global multicenter study coordinated by C‐RIDL, we set out to establish a population‐based RIs for hematological parameters, including serum iron markers, and related vitamins for healthy Ghanaian adults based on the international harmonized protocol and utilizing advanced statistical methods.

## 2 MATERIALS AND METHODS

### 2.1 Volunteer recruitment

Apparent healthy volunteers were recruited in accordance with the protocol published by the IFCC, Committee on Reference Intervals and Decision Limits (C‐RIDL), for multicenter studies.^12^ The reference individuals were recruited from public institutions, schools, health facilities, social centers, churches, and mosques from June to December 2017. A total of 501 study participants aged 18‐70 years from the Greater Accra and Northern regions in Ghana representing different ethnic groups were recruited. The sample size was appropriately computed as to be adequate and to guarantee reproducible test results for making between‐country comparison.^12^

Structured questionnaires were administered to volunteers to collect data on demographics, lifestyles, nutrition pattern, and health‐status. The selection of eligible participants was based on well‐defined inclusion and exclusion criteria, in accordance with the IFCC/C‐RIDL protocol.^12^ The exclusion criteria included the following: individuals with known diabetes mellitus under drug therapy, recent (≤14 days) recovery from acute illness, injury, or surgery requiring hospitalization, known carrier state of hepatitis B virus, hepatitis C virus, or HIV, pregnancy or within one year after childbirth and incomplete data (questionnaire/consent form not completed). However, considering a Ghanaian general standard of healthiness, additional exclusion criteria not stipulated in the C‐RIDL protocol were set to exclude individuals with BMI ≥ 35 kg/m^2^, consumption of alcohol ≥70 g/d, smoking habit of >20 cigarettes/d.

Upon completion of the questionnaire and consent form, eligible volunteers in Accra were then scheduled for collection of fasting venous blood at Medlab Ghana Ltd. For volunteers in Tamale, blood was drawn on site and air transported to Medlab Ghana in Accra within 6 hours each day for processing.

### 2.2 Laboratory analysis

#### 2.2.1 Blood sampling and storage

Blood drawing was done under basal conditions as recommended in the IFCC/C‐RIDL protocol between 7:00‐10:00 am after overnight fasting for 10‐14 hours, avoidance of strenuous muscular exertion for three prior days, sitting still for at least 20 minutes prior to venipuncture to avoid postural changes.^20^ A fasting blood sample of 2 mL was drawn into two plastic vacutainer tubes containing ethylenediaminetetraacetic acid (EDTA) (BD Vacutainer® Blood Collection Tube, West Africa) for complete blood count (CBC). Subsequently, to ensure uniformity and prevent coagulation, each sample tube was immediately inverted gently 3 ~ 4 times. Also, 9 mL fasting blood samples were drawn into BD Vacutainer® SST tubes (Becton‐Dickinson Corp) containing a clot‐activator. Analytes tested in serum were the non‐CBC parameters listed below. Anthropometric measurements (weight, height, waist circumference, blood pressure) were taken on the day of sample collection.

#### 2.2.2 Analytical procedure

Whole blood samples were analyzed for CBC within 6 hours after sample collection using a Sysmex XN‐1000 analyzer (Sysmex Corp). Hematological analytes included in the CBC were red blood cells (RBC), hemoglobin (Hb), hematocrit (Hct), mean corpuscular volume (MCV), mean corpuscular hemoglobin concentration (MCHC), red cell distribution width (RDW), platelet (PLT), mean platelet volume (MPV), white blood cells (WBC) and its differentials‐neutrophils (Neu), eosinophils (Eos), lymphocytes (Lym), basophils (Bas), monocytes (Mon). The differentials were recorded as both percentage (%) and absolute counts. Immunoglobulins G, A, and M (IgG, IgA, IgM), complement component 3 (C3), folate, transferrin (TF), and vitamin B12 (VitB12) were analyzed using Centaur XP Siemens' analyzer (Bayer, Germany), while iron (Fe), ferritin (Ferr), and C‐reactive protein (CRP) were analyzed using Beckman‐Coulter AU 480 analyzer (Beckman‐Coulter). All the analytes were conducted in Medlab Ghana, an ISO 15189 accredited Medical Laboratory which permitted high‐quality and reproducible results.

The serum specimens for non‐hematological parameters (IgG, IgA, IgM, C3, C4, CRP) were centrifuged after sampling to separate the serum daily. The sera were stored at −80°C until the time of collective measurements. Those analytes were measured together with other analytes for a parallel study for establishing chemistry/immunology RIs. Test results for a part of chemistry analytes were used in this study as a part of reference tests in the latent abnormal values exclusion (LAVE) procedure (see below) for reducing the influence of individuals with latent anemia or inflammation.

#### 2.2.3 Quality control

All laboratory investigations were carried out in accordance with the laboratory's Quality Manual and standard operating procedures (SOPs). We adhered to instructions described in each analyser's manual and reagent/kit package inserts. During the period of collective measurements of non‐hematological parameters in batches of 80 ~ 100 specimens, between‐day quality control was performed by testing mini‐panel of sera as specified in IFCC/C‐RIDL protocol.^12^

### 2.3 Ethical approval

The study protocol was approved by the Ethical Review Committee (CHS‐Et/M.8‐P 4.14/2016‐2017) of the College of Health Sciences, University of Ghana. Informed consent was obtained from all eligible volunteers.

### 2.4 Statistical analyses

#### 2.4.1 Sources of variation (SV) of reference values

Multiple regression analysis (MRA) was performed separately for males and females by setting RVs of each analyte as an objective variable. Explanatory variables, age, BMI, ethnicity (Akan = 1; others = 0), Systolic BP, and Diastolic BP were set constant. The degree of association of each explanatory variable with the objective variable was expressed as a standardized partial regression coefficient (rp), which takes a value between −1.0 and 1.0. We considered |rp| ≥ 0.2 as an appreciable effect size between small (0.1) and medium (0.3) correlation specified by Cohen.^21^

#### 2.4.2 Partitioning criteria for reference values

The need for partitioning RVs by sex and age was judged by standard deviation ratio (SDR). The SDR represents a ratio of between‐subgroup SD (variation of the subgroup means from grand mean) to between‐individual SD (approximately 1/4 the width of RI, representing between‐individual SD). Two‐level nested ANOVA was performed to compute between‐sex SD and between‐age group SD after partitioning age as 18‐29, 30‐39, 40‐49, and 50+ years. The SDR for between‐sex SD (SDR_sex_) and for between‐age SD (SDR_age_) was computed as a ratio to residual SD (or between‐individual SD). Because between‐age variation changes by sex, one‐way ANOVA was also performed to compute SDR_age_ for each sex. The SDR ≥ 0.40 was considered as practically significant between‐subgroup difference to derive sex‐ or age‐specific RIs.^13^, ^22^

However, SDR is sometimes insensitive to actual difference (bias) at LL or UL after partitioning because SDR represents central tendency of variation by the SV, not necessarily represents variation in the periphery. Therefore, we set up an additional index called bias ratio (BR) at LL or UL, BR_LL_, or BR_UL_, to cope with the problem.

$${\text{BR}}_{\text{LL}}=\frac{\left|{\text{LL}\;}_{M}‐{\text{LL}}_{F}\right|}{\left({\text{UL}}_{\text{MF}}‐L_{\text{MF}}\right)/3.92},\;{\text{BR}}_{\text{UL}}=\frac{\left|{\text{UL}}_{M}‐{\text{UL}}_{F}\right|}{\left({\text{UL}}_{\text{MF}}‐{\text{LL}}_{\text{MF}}\right)/3.92},$$

where subscript M, F, and MF represent male, female, and male + female, respectively.

In accordance with the convention of allowable bias^23^ specification of a minimum level: 0.375 × SD_G_ (=SD_RI_), we regard BR_UL_ > 0.375 as an auxiliary criterion for partitioning RVs when SDR does not match to actual between‐subgroup difference at ULs (or LLs).

#### 2.4.3 Derivation of reference intervals

Parametric and nonparametric methods were used in deriving the RIs. For the parametric method, the reference values were first transformed into Gaussian distribution by use of the modified Box‐Cox power transformation formula^13^, ^24^, in determining the mean and SD, the final RI was calculated as the mean ± 1.96 SD (after truncating the values outside mean ± 2.81 SD once), which corresponds to the lower and higher limits (LL and UL) of the RI under the transformed scale. Then, the limits were reverse transformed to get the LL and UL in the original scale.

The 90% confidence intervals (CIs) for both LL, UL of the RI were estimated by use of the bootstrap method through iterative resampling 50 times. Making use of this procedure, the final RIs were set to averages of LL and UL computed repeatedly.

One important aspect of the data analysis was to detect and remove inappropriate values that represent latent diseases of common occurrence. Therefore, prior to derivation of RIs, the LAVE method was used to exclude subjects with latent disease conditions/abnormal values. The LAVE method is an iterative optimization method for filtering reference individuals by excluding subjects with abnormal values in related analytes.^13^, ^24^ In the initial calculation, RIs were calculated test by test independently without any exclusion, but in the subsequent iterative calculation, any individual who had two or more results outside the previously derived RIs among the reference tests (described below) were excluded.

The RIs were computed in two parts: one for markers of erythropoiesis and iron metabolism (folate, VitB12, Fe, TF, ferritin, RBC, Hb, Ht, MCV, MCH, MCHC, and RDW), the other for leukocytes and platelets (WBC, differential counts, PLT, and MPV). Reference tests used for the erythrocytes group were Fe, TF, ferritin, Hb, Ht, and MCV, and for the leukocytes group were IgG, IgA, IgM, C3, CRP, and ferritin.

The BR_LL_ or BR_UL_ was also used for judging the need for the LAVE method by the following equation.

$${\text{BR}}_{\text{LL}}=\frac{\left|{\text{LL}\;}_{\text{LAVE}+}‐{\text{LL}}_{\text{LAVE}\;‐\;}\right|}{\left({\text{UL}}_{\text{LAVE}\;+\;}‐{\text{LL}}_{\text{LAVE}+}\right)/3.92},\;{\text{BR}}_{\text{UL}}=\frac{\left|{\text{UL}\;}_{\text{LAVE}\;+\;}‐{\text{UL}}_{\text{LAVE}‐}\right|}{\left({\text{UL}}_{\text{LAVE}\;+\;}‐{\text{LL}}_{\text{LAVE}\;+\;}\right)/3.92}.$$

## 3 RESULTS

### 3.1 Demographic profile of the participants

The total sample size of reference individuals included in data analysis was 501, comprising 54% (n = 270) males and 46% (n = 231) females. The participants' mean age and standard deviation (SD) were 41.3 ± 13.5 years with the following categories: 18‐29 years (24.7%), 30‐39 years (22.5%), 40‐49 years (23.9%), and ≥50 years (28.8%). 77.2% of the volunteers were from Accra and 22.8% from Tamale. With regards to ethnicity, Akan constituted 40.3% (202) and non‐Akans were 59.7% (299). Mean BMI was 25.8 ± 3.9 kg/m^2^ (range: 18.6‐34.9 kg/m^2^). With regards to blood pressure, the mean systolic values were 121.2 ± 11.5 mm Hg (range: 88‐140 mm Hg) and diastolic values were 78.9 ± 7.2 mm Hg (range 60‐90 mm Hg), respectively. Almost all participants (99.4%) were non‐smokers. Based on the questionnaire, separating allergic symptoms into pollinosis, asthma, or atopic dermatitis, only 5.2% of males and 5.2% of females had experienced an allergic symptom, this result was relevant for interpreting leukocyte counts, especially for Eosinophils. Seven individuals (1.4%) reported taking food supplements (folate 3, iron 1, multivitamin 2, miscellaneous 1). However, we did not exclude them in the analysis of corresponding analytes as we regarded this as having negligible influences.

### 3.2 Sources of variation and the scheme for partitioning reference values

The MRA results are shown in Table S1. By regarding the effect size (practical significance) of rp as 0.20, blood pressure had no association with RVs of any analyte except VitB12 (rp = 0.26) in males, and PLT in females (rp = 0.20). Age‐related increase was noted for RVs of ferritin (rp = 0.44) and folate (rp = 0.24) among females, while in males, age‐related decrease was observed in RVs of Hb, RBC, Ht with rp of −0.20, −0.33, and −0.27, respectively. For ethnicity, folate RVs were higher in Akans compared to non‐Akans for both sexes (males rp = 0.21 and female rp = 0.24). Also, the level of MCHC was higher in Akan males compared to that of non‐Akan males (rp = 0.24). There was a positive association between BMI and RVs for ferritin in males (rp = 0.30) and eosinophils in females (rp = 0.21).

Using two‐level nested ANOVA, between‐sex differences (SDR_sex_ ≥ 0.4) were observed for six analytes (RBC, Hb, Ht, Fe, Ferr, and PLT) as shown in Table 1. Also, age‐related decrease of RVs with SDR ≥ 0.4 was noted for RBC (SDR_age_ = 0.42) in males and TF (SDR_age_ = 0.41) in females, while increase of RVs by age was noted for only ferritin (SDR_age_ = 0.56) in females (Table 1).

**TABLE 1:** Standard deviation ratios for the magnitude of sex and age changes for hematological analytes

Analyte	SDR_sex_	SDR_age_M	SDR_age_F
WBC	0.085	0.141	0.128
MPV	0.030	0.164	0.061
PLT	**0.455**	0.000	0.291
Neu %	0.090	0.028	0.061
Neu	0.134	0.085	0.082
Lym %	0.000	0.125	0.115
Lym	0.091	0.101	0.111
Mon %	0.207	0.000	0.000
Mon	0.024	0.000	0.114
Eos %	0.206	0.000	0.000
Eos	0.153	0.075	0.000
Bas %	0.066	0.040	0.000
Bas	0.000	0.143	0.000
Fe	**0.534**	0.000	0.076
Ferr	**0.723**	0.271	**0.559**
TF	0.111	0.241	**0.409**
VitB12	0.233	0.000	0.000
Folate	0.187	0.062	0.226
RBC	**1.168**	**0.421**	0.000
Hb	**1.451**	0.224	0.000
Ht	**1.362**	0.256	0.000
MCV	0.000	0.287	0.095
MCH	0.103	0.293	0.000
MCHC	**0.376**	0.218	0.000
RDW	0.282	0.000	0.000

Note

Two‐level nested ANOVA was used to compute between‐sex SD (SD_sex_) and between‐age SD (SD_age_) and between‐individual SD (SD_bi_). SDR_sex_ was derived as SD_sex_/SD_bi_. For deriving sex‐specific SDR_age_ (SDR_age_M, and SDR_age_F), one‐way ANOVA was performed separately for each sex. Values of SDRs ≥ 0.3 are shown in bold and SDRs ≥ 0.5 were highlighted by gray background.

**FIGURE 1:**
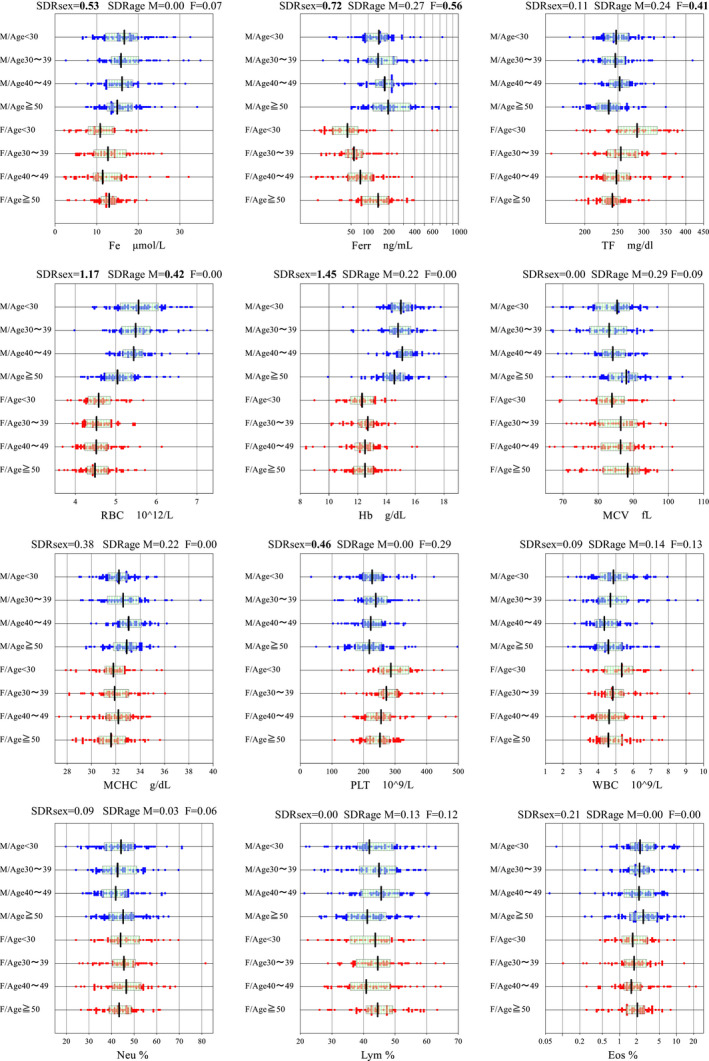
Sex‐ and age‐related changes in RVs for 8 or 12 representative hematological analytes. All values (RVs) were plotted without any exclusion after stratification by sex and age (in four strata). The blue and red dots represent values of males (M) and females (F), respectively. The box in the center represents central 50% range and the vertical line in its center indicates a median point. The SDR_sex_ was derived by two‐level nested ANOVA and SDR_age_ by one‐way ANOVA. SDR ≥ 0.4 was marked by bold font

The graphical representation of sex‐ and age‐related changes for all the hematological parameters are shown in Figure S1. In Figure 1, the graphs are shown for 8 or 12 selected analytes with conspicuous sex and/or age‐related variations.

### 3.3 Derivation of reference intervals

**FIGURE 2:**
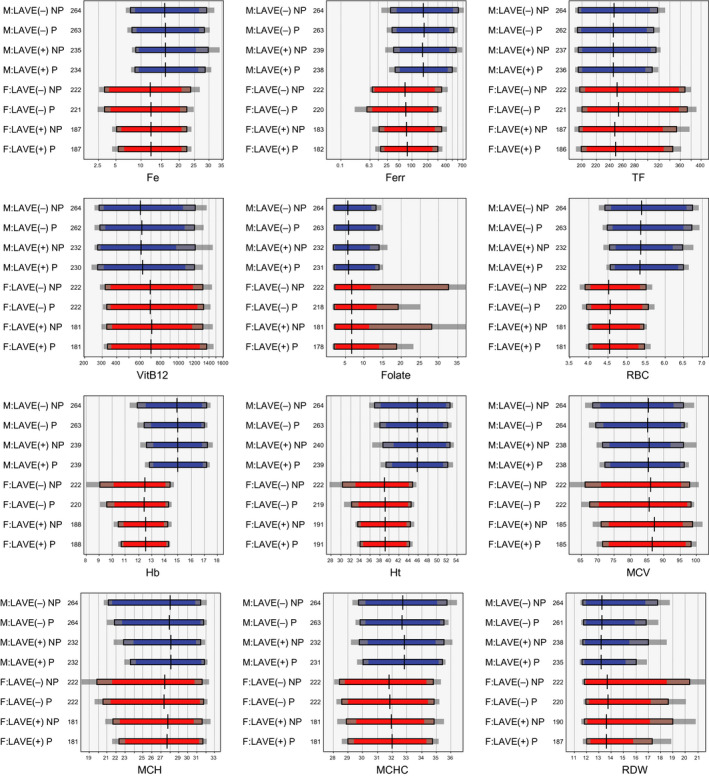
Comparison of RIs derived by parametric and nonparametric methods with or without latent abnormal values exclusion (LAVE) method. RIs were derived by use of four calculation methods: parametric (P) and nonparametric (NP) methods with/without LAVE. Reference tests used for LAVE for the analytes shown were Fe, TF, Ferr, Hb, Ht, and MCV. The colored bars (blue for males and red for females) represent the span of lower and upper limits of RIs. The gray shade at both ends of the bars represents 90% confidence intervals

We derived the RIs in four ways by parametric (P) and nonparametric (NP) method with/without LAVE. The comparisons of RIs for twelve selected analytes are shown in Figure 2. It was obvious that 90% confidence limits for LL and UL were generally wider by use of NP method, as documented elsewhere.^11^, ^13^, ^15^ The LAVE method had an obvious effect in raising LLs of Fe, Ferr, RBC, Hb, Ht, MCV, and MCH and in lowering ULs of TF and RBC. This prominent LAVE effect was obviously reflecting successful exclusion of individuals with latent anemia, although the method caused approximately 10% and 15% reduction in data size for males and females, respectively.

In Table S2, a full comparison of RIs by the P method with/without LAVE is shown for all the analytes. The effect of the LAVE method on LL or UL was judged significant when BR_LL_ or BR_UL_ exceeded the threshold of 0.375. This was true for most of the parameters related to erythrocytes and iron: RBC, Hb, Ht, MCV, MCH, RDW, Fe, Ferr, and TF. For the parameters related to leukocytes and platelets, the LAVE method (using inflammatory markers as the reference tests) had effect only for Neu# and Eos# with lowering of their ULs.

Table 2 presents the list of RIs for hematological and related analytes, which were derived using parametric method in three ways: males + females, males, and females. For the selection of RIs with/without partition by sex and age, we primarily relied on SDR_sex_ and SDR_age_, but we considered the bias at the RI limits (BR_LL_ and BR_UL_), which are listed in Table S2.

**TABLE 2:** Reference intervals for hematological analytes derived by parametric method

Item	Unit	LAVE	Sex	Age	n	90% CI of LL		Reference intervals			90% CI of UL	
								LL	Me	UL		
RBC	10^12^/L	(+)	M	18 ~ 70	232	4.45	4.68	4.57	5.34	6.50	6.35	6.64
		(+)	F	18 ~ 70	181	3.90	4.09	4.00	4.54	5.46	5.31	5.62
Hb	g/L	(+)	M	18 ~ 70	239	12.5	13.1	12.8	15.0	17.2	16.9	17.4
		(+)	F	18 ~ 70	188	10.4	10.9	10.7	12.6	14.3	14.1	14.4
Ht	%	(+)	M	18 ~ 70	239	38.1	40.6	39.4	45.8	52.1	51.1	53.2
		(+)	F	18 ~ 70	191	33.3	34.6	34.0	39.1	44.2	43.6	44.9
MCV	fL	(+)	MF	18 ~ 70	428	70.4	73.3	71.9	86.0	97.4	96.6	98.2
MCH	pg	(+)	MF	18 ~ 70	419	22.7	23.8	23.2	28.1	31.8	31.6	32.0
MCHC	g/L	(−)	MF	18 ~ 70	485	28.7	29.4	29.0	32.3	35.2	35.0	35.5
RDW	%	(+)	M	18 ~ 70	235	11.5	11.9	11.7	13.2	16.0	15.2	16.9
		(+)	F	18 ~ 70	187	11.7	12.4	12.0	13.6	17.3	15.8	18.9
Fe	µmol/L	(+)	M	18 ~ 70	234	7.8	9.4	8.6	16.1	28.5	26.3	30.7
		(+)	F	18 ~ 70	187	4.4	6.5	5.5	12.4	22.4	20.8	24.0
Ferr	µg/L	(+)	M	18 ~ 70	238	27	51	39	168	502	431	573
		(+)	F	18 ~ 70	182	10	20	15	79	300	244	357
TF	mg/dL	(+)	M	18 ~ 70	236	191	200	195	245	308	297	319
		(+)	F	18 ~ 70	186	192	207	199	248	345	329	362
VitB12	pmol/L	(−)	M	18 ~ 70	262	256	322	289	611	1199	1073	1326
		(−)	F	18 ~ 70	222	308	357	332	691	1330	1238	1422
Folate	pmol/L	(−)	M	18 ~ 70	263	1.8	2.4	2.1	5.9	14.1	13.2	15.0
		(−)	F	18 ~ 70	218	1.6	2.4	2.0	6.7	19.3	13.4	25.1
WBC	10^9^/L	(−)	MF	18 ~ 70	483	2.84	3.31	3.08	4.75	7.53	7.15	7.91
MPV	fL	(−)	MF	18 ~ 70	475	8.93	9.27	9.1	10.7	12.8	12.58	12.93
PLT	10^9^/L	(−)	M	18 ~ 70	262	100	130	115	228	339	323	356
		(−)	F	18 ~ 70	222	145	169	157	268	402	381	424
Neu %	%	(+)	MF	18 ~ 70	427	27.6	29.4	29	44	62	59.9	63.4
Neu #	10^9^/L	(+)	MF	18 ~ 70	413	1.13	1.21	1.17	2.03	3.81	3.63	3.98
Lym %	%	(−)	MF	18 ~ 70	485	25.0	28.0	27	44	60	58.5	60.7
Lym #	10^9^/L	(−)	MF	18 ~ 70	485	1.16	1.28	1.22	2.01	3.38	3.28	3.49
Mon %	%	(−)	MF	18 ~ 70	485	4.72	5.30	5.0	8.5	13.7	13.18	14.15
Mon #	10^9^/L	(−)	MF	18 ~ 70	484	0.21	0.24	0.22	0.40	0.73	0.68	0.77
Eos %	%	(−)	M	18 ~ 70	262	0.30	0.60	0.5	2.4	10.3	8.39	12.21
		(−)	F	18 ~ 70	219	0.28	0.47	0.4	1.8	6.5	5.49	7.53
Eos #	10^9^/L	(+)	M	18 ~ 70	209	0.02	0.03	0.03	0.12	0.53	0.41	0.64
		(+)	F	18 ~ 70	188	0.01	0.03	0.02	0.09	0.29	0.25	0.34
Bas %	%	(−)	MF	18 ~ 70	485	0.16	0.21	0.2	0.6	1.5	1.33	1.57
Bas #	10^9^/L	(−)	MF	18 ~ 70	485	0.01	0.01	0.01	0.03	0.08	0.07	0.08

Note

The RIs we adopted were indicated by values in bold font. The symbol (+) at the LAVE column in the table indicates that RI by the LAVE procedure was adopted based on BiasLL and BiasUL shown in Table S2. # indicates absolute counts, while % depicts absolute percentage. No retriction of RVs by age was applied.

Abbreviations: LL, lower limit of RI; Me, median; UL, upper limit of RI.

For partitioning of RVs by sex, we chose RBC, Hb, Ht, Fe, Ferr, and PLT based on SDR_sex_ ≥ 0.4. Whereas, RVs for RDW, TF, VitB12, folate, and Eos had SDR_sex_ below 0.4, but actual between‐sex difference at LL or UL expressed as BR_LL_ or BR_UL_ was high (>0.375), and thus we chose to partition their RIs by sex.

According to these schemes for partitioning, the final RIs we adopted were shown by values in bold fonts. Although partition of RIs by age at 45 years was found necessary from SDR_age_F for Ferr and TF of females, it was not applied because of too small data size for the 45+ age statum (n<100).

**FIGURE 3:**
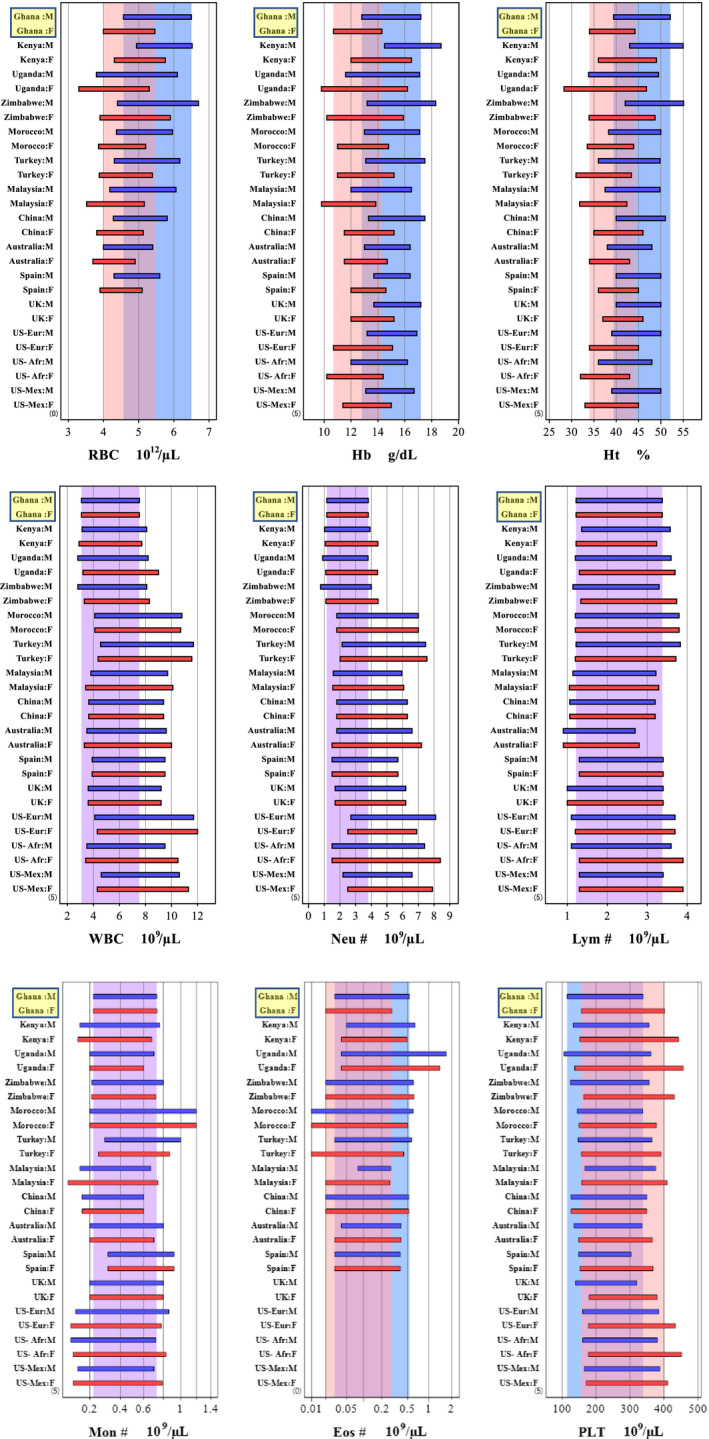
Graphical comparison of Ghanaian reference intervals (RIs) for hematological parameters with those reported in other countries. The pair of horizontal bars for each study represent RIs for male (M) in blue, and female (F) in red. The background shades in blue and pink represent RIs of this Ghanaian study for M, and F, respectively. Eos#, eosinophil counts; Hb, hemoglobin; Ht, hematocrit; Lym#, lymphocyte counts; Mon#, monocyte counts; Neu#, neutrophil counts; PLT, platelet count; RBC, red blood cells; WBC, white blood cell count

The between‐country comparison graphs are shown for CBC parameters in Figure 3. For erythrocyte parameters, ULs of RBC, Hb, and Ht in Ghanaian males were higher than most of other countries except Kenya and Zimbabwe, where volunteers were recruited from high altitude areas. On the contrary, the UL of Hb in Ghanaian females was lowest among the 14 countries examined except Malaysia. The LL of RBC, Hb, and Ht in Ghanaian females was amongst the lowest in the world but higher than those derived for Uganda, Zimbabwe Morocco US‐African descent, and Malaysia.

For leukocyte parameters, there was a prominent trend of low levels of WBC and Neu# among the African countries except Morocco. By focusing on Ghanaian trend, it is clearly noted that the UL of WBC for both males and females was the lowest among the African countries studied. Besides, the width of RIs for Neu# is narrower compared to all other countries.

With regards to Eos#, the Ghanaian RIs were appreciably lower, especially in females, compared to all the African countries, reflecting very low prevalence of allergic diathesis in the Ghanaian population recruited for the study (Section 3.1).

For platelets, the RI for Ghanaian males was shifted to a lower side compared to most of other countries. Also, the UL of Ghanaian females for platelet is higher than most of the non‐African countries.

Further, we compared our CBC RIs with three other studies conducted in Ghana, all of which used the nonparametric method without any procedure for secondary exclusion. As shown in Figure S2, with regard to erythrocyte parameters, LLs for RBC, Hb, and Ht of our study were conspicuously higher than other studies both in males and females. For the leukocyte parameters, there was a noticeable trend of lower levels of our ULs for WBC, Neu#, Lym#, Mon#, and Eos# than other studies, with much narrower width of the RIs. On the other hand, the LLs of platelets in our study were generally higher in both males and females.

## 4 DISCUSSION

Proper interpretation of RVs requires understanding the sources of variation that could influence laboratory tests such as sex, age, ethnicity, and BMI. Detailed evaluation of these factors was an important part of this study, which provided a clue for the need for partitioning or exclusion of RVs. We used SDR as a measure to quantify the magnitude of between‐sex and age differences. By setting SDR = 0.4 as its threshold, we observed significant between‐sex differences in RVs for many erythrocyte and iron parameters: RBC, Hb, Ht, RDW, Fe. We compared our results with Kenya,^11^ Japan,^25^, ^26^, ^27^ and Turkey,^16^ and found that our SDR_sex_ values are consistent with these countries.

Besides the parameters of erythrocyte and iron metabolism, between‐sex difference was noted only for PLT (SDR_sex_ = 0.46). This finding is consistent with Kenyan's result (SDR_sex_ = 0.41),^11^ but contradicts what was reported in Japan (SDR_sex_ = 0.13 ~ 0.23),^27^ and Turkey (SDR_sex_ = 0.23).^16^ This gender difference of RIs for PLT, females: 157‐402 × 10^9^/L vs males: 115‐339 × 10^9^/L has been attributed to the effects of endogenous female sex hormones, mainly the estrogen, on the activation of platelet formation.^28^, ^29^ It may also be due to reactive causes of thrombocytosis such as bleeding from any cause especially menorrhagia in females. Interestingly, Mon% was found to require sex‐specific RIs according to what Omuse et al^11^ found among Kenyans, but it is less obvious in our study.

As a special finding of gender difference, we encountered five special analytes, RDW, TF, VitB12, folate, and Eos that showed SDR_sex_ well below 0.4, but actual between‐gender difference at LL or UL was high if computed as Bias_LL_ or Bias_UL_ (>0.375). This phenomenon indicates that SDR represents between‐group differences at the center of the distribution, and thus, even if SDR is small, between‐sex difference at LL or UL can be large enough to warrant partition of RVs by sex.

With respect to the age‐related changes, RBC showed age‐related decrease with SDR_age_ = 0.42 among males, which was in contrast with other studies conducted in Kenya,^11^ Japan,^26^, ^27^ and Turkey.^16^ This finding in the Ghanaian population may be due to dietary factors, since Ghanaian meals are high in carbohydrates and low in protein or due to unknown genetic predisposition which could result in a decrease in erythropoietin levels in older individuals.^30^, ^31^ Similar to other studies,^11^, ^16^ we observed conspicuous age‐related increase in Ferr and decrease in TF in females (Figure 1). Low Ferr levels in women are obviously attributed to reduced iron stores due to menstrual bleeding and pregnancy. The age‐related increase in Ferr levels occurring in women confirms a study reported by Kato et al^32^ which found that Ferr concentrations in postmenopausal women were twice as high as premenopausal women. Consistent with previous studies,^33^, ^34^ the current study also found folate levels to be higher in women than men with no age‐related variation.

RIs of Hb and Ht for Ghanaian males and females were similar to those reported in recent studies from Kenya, Japan, Turkey, except that in Kenyans, they were significantly shifted to the higher side, which could be attributable to the high altitude of Nairobi.^11^ One of the features of Ghanaian hematological RIs was the low UL of Hb among Ghanaian females compared with other SSA countries and their western counterparts as shown in Figure 3. The low levels of Hb among females in our study may be multifactorial. Foremost, the low levels of dietary bioavailable iron and secondly, the fact that majority of our female participants were of the reproductive age group may have contributed to the low Hb levels.

A comparison of leukocyte parameters reveals that RIs for WBC and neutrophil counts in some SSA countries (Kenya, Uganda, and Zimbabwe)^10^, ^11^, ^35^ were lower compared to the other countries. This observation is well‐known, but its etiology is still not established although previous studies have reported that the low WBC and neutrophil counts among African ancestry are associated with the common African‐derived null variant (rs2814778) of the Duffy antigen receptor for chemokines (DARC‐null genotype).^36^, ^37^ This gene is known to make Africans less vulnerable to Plasmodium Vivax infections.^37^ The current study observed higher UL of Lym# compared with those reported from Malaysia,^38^ China,^39^ and Australia.^40^ The effect of chronic antigenic stimulation may account for this observation and probably the higher prevalence of parasitic infections among Ghanaians.^11^, ^19^, ^41^

We also observed that in our population, Eos# levels were higher compared with Spain, Australia, and Malaysia^38^, ^40^, ^42^ but were relatively lower compared with other African countries.^10^, ^11^, ^35^ A generally higher prevalence of parasitic infestation in African countries and a very low prevalence of allergic diseases in our study population (5.2% in both sexes) may explain these observations. Furthermore, RI derived for lymphocytes is consistent with the values reported in Kenya and the SSA region,^11^ but relatively higher than those reported in Turkey^16^ and Japan.^27^

It is also noteworthy that neutrophil counts are much higher than lymphocyte counts in Turkey, Malaysia, Australia, and UK^16^, ^38^, ^40^, ^43^ while both counts are approximately the same in Ghana and other African countries.^10^, ^11^ No clear explanation is available, but we assume that immunological surveillance system depends on environmental and/or genetic factors. Our study also showed that folate levels were lower compared with those reported in Asian study^22^ and in the interim report of the IFCC global study.^15^ This observation may be due to nutritional factors such as low intake of green vegetables among Ghanaians, since most of Ghanaian meals are carbohydrate and cereal based.

Interestingly, comparison of our RIs with three other studies conducted in Ghana revealed significant differences between them as shown in Figure S2. The LLs of most erythrocyte parameters in our study are higher: for example, we obtained 10.8 g/L as LL of Hb for female, while Study‐2, −3, and −4 reported 8.8,^18^ 8.2,^19^ and 9.1 g/dL,^17^ respectively. Similarly, our RIs for WBC and its differential (Neu, Eos, and Mon) were generally shifted to the lower side.

These discrepancies are probably attributable to improved methodological approaches that we employed, especially (a) the use of parametric method which is generally robust to outliers than the nonparametric method recommended by the CLSI guideline, and (b) the application of the LAVE method that effectively reduced the influence of prevalent subclinical conditions: that is, latent anemia for Hb, and subclinical inflammation for leukocyte counts.

The effects of both approaches are clearly shown in Figure 2, which demonstrated differences in the RIs derived by parametric or nonparametric, with/without LAVE. The findings indicate the robustness and higher precision of the parametric method, that is,, narrower 90% CI of RI limits,^11^, ^13^, ^24^ and the efficiency of LAVE method. In other words, even if careful elaboration of a priori selection criteria was implemented to exclude inappropriate volunteers during recruitment, it is still possible to include a sizable number of participants with latent diseases.^13^, ^25^, ^44^, ^45^, ^46^ Therefore, we believe it is essential to apply the LAVE method in deriving hematological RIs.

In summary, a major strength of this study was the implementation of stringent schemes for conducting the study both in the recruitment of well‐defined healthy volunteers based on IFCC/C‐RIDL protocol, and in the use of robust statistical methods including the LAVE method. The scheme certainly helped us to increase the precision of calculating the RI, and to reduce the influence of latent anemia and inflammation. On the other hand, a limitation of this study was that even though the volunteers' background is nationally representative in terms of ethnicity, the results represent the urban Ghanaian and may not reflect healthy Ghanaian adults who live in rural settings with different lifestyle in terms of food, occupation, and physical activities.

## 5 CONCLUSION

This study confirmed the advantage of the parametric method over the nonparametric method and the need for application of the LAVE method for analytes, which are easily influenced by high prevalence of conditions such as latent anemia and inflammations. Based on the objective criteria (SDR and BR), partitioning RVs by sex was shown to be necessary not only for erythrocyte parameters, but also for iron markers, PLT, and Eos#. It is of note that the Ghanaian RIs for WBC and Neu# were markedly low compared to those of non‐African countries. The low RI for Hb for the female Ghanaian even after the LAVE procedure is attributable to generally low iron reserves.

The robust statistical techniques used in this study made the RIs well matched to Ghanaian population. We hope the hematological RIs from this study will be adopted in regional laboratories and thereby lead to improved clinical decision‐making and healthcare in Ghana and the surrounding countries.

## CONFLICT OF INTEREST

The authors have no competing interests.

## AUTHORS CONTRIBUTIONS

Kiyoshi Ichihara, Rajiv Erasmus, Rosemary Keatley, Patrick F. Ayeh‐Kumi, Julius Fobil, John Arko‐Mensah, and Abigail S.A. Bawua contributed to the conception and designed of the project. Research investigations were conducted by Abigail S.A. Bawua, Rosemary Keatley, Patrick F. Ayeh‐Kumi, Julius Fobil, John Arko‐Mensah, and Yvonne Dei‐Adomakoh. Data collection, analysis and interpretation were performed by Kiyoshi Ichihara and Abigail S.A. Bawua. The initial draft of the manuscript was written by Abigail S.A Bawua, Kiyoshi Ichihara Julius Fobil, and Yvonne Dei‐Adomakoh with critical review by John Arko‐Mensah, Rajiv Erasmus, Patrick F. Ayeh‐Kumi, and Rosemary Keatley. Kiyoshi Ichihara, Julius Fobil, Patrick F. Ayeh‐Kumi, Rosemary Keatley, John Arko‐Mensah, and Yvonne Dei‐Adomakoh supervised the findings of this research. All authors discussed the results and contributed to the final manuscript write up.

## Supporting information

Appendix S1
