# Supplementary material for: Establishing Ghanaian adult reference intervals for hematological parameters controlling for latent anemia and inflammation
Source: Int J Lab Hematol. 2020 Sep 3;42(6):705–17. doi: 10.1111/ijlh.13296 (PMC7754426; doi:10.1111/ijlh.13296)
Supplement: Supplementary file 1 — Appendix S1 [file IJLH-42-705-s001.docx]

**Suppl. Table 1. MRA results (r_p_) for sources of variation of reference values in males and females**

 Standardized partial regression coefficient (r_p_); r_p_ ≥ 0.20 was considered significant. All numbers bolded indicates significance level with r_p_ ≥ 0.20.

**
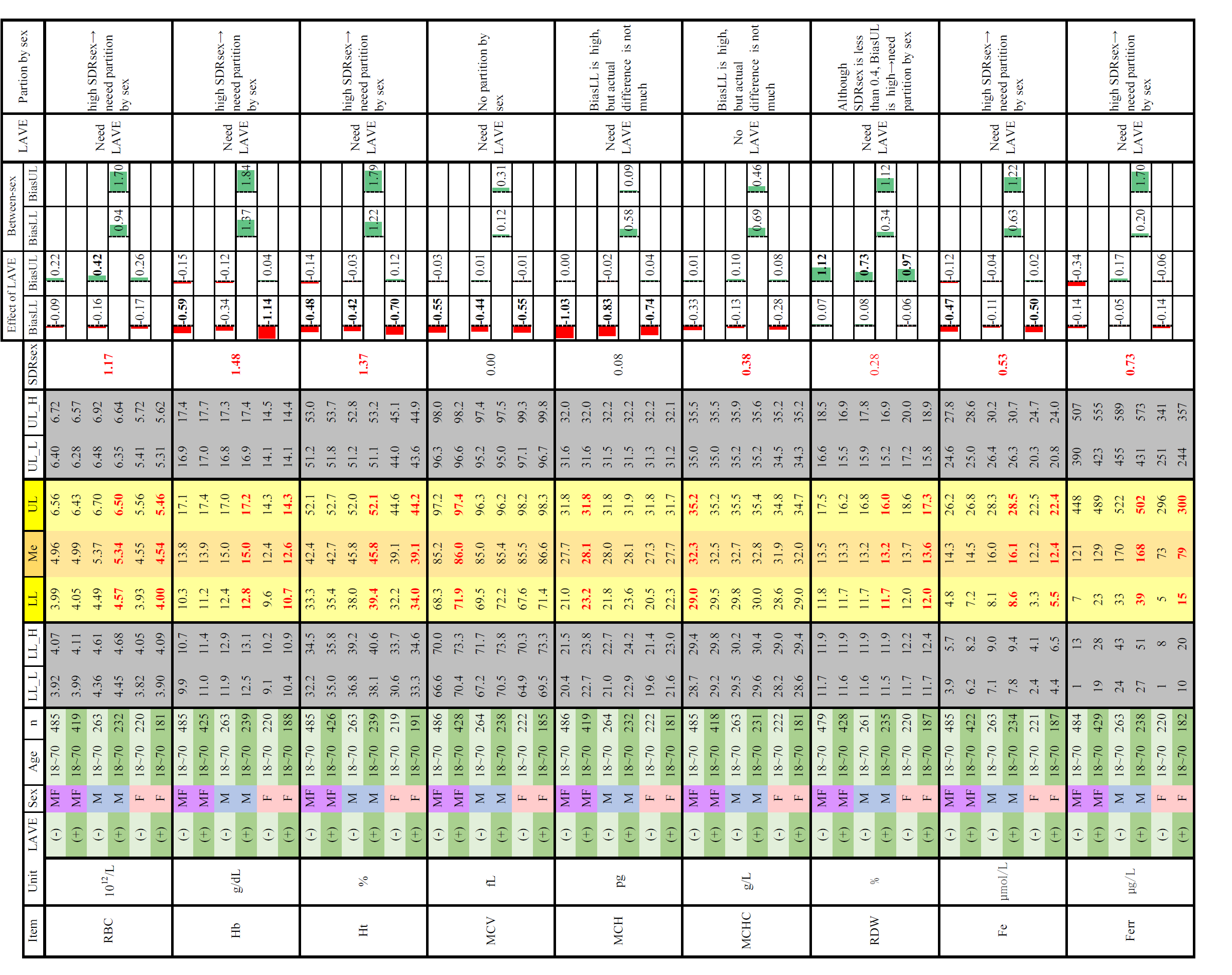
Suppl. Table 2: Full list of RIs partitioned by sex with/without the LAVE method**

**
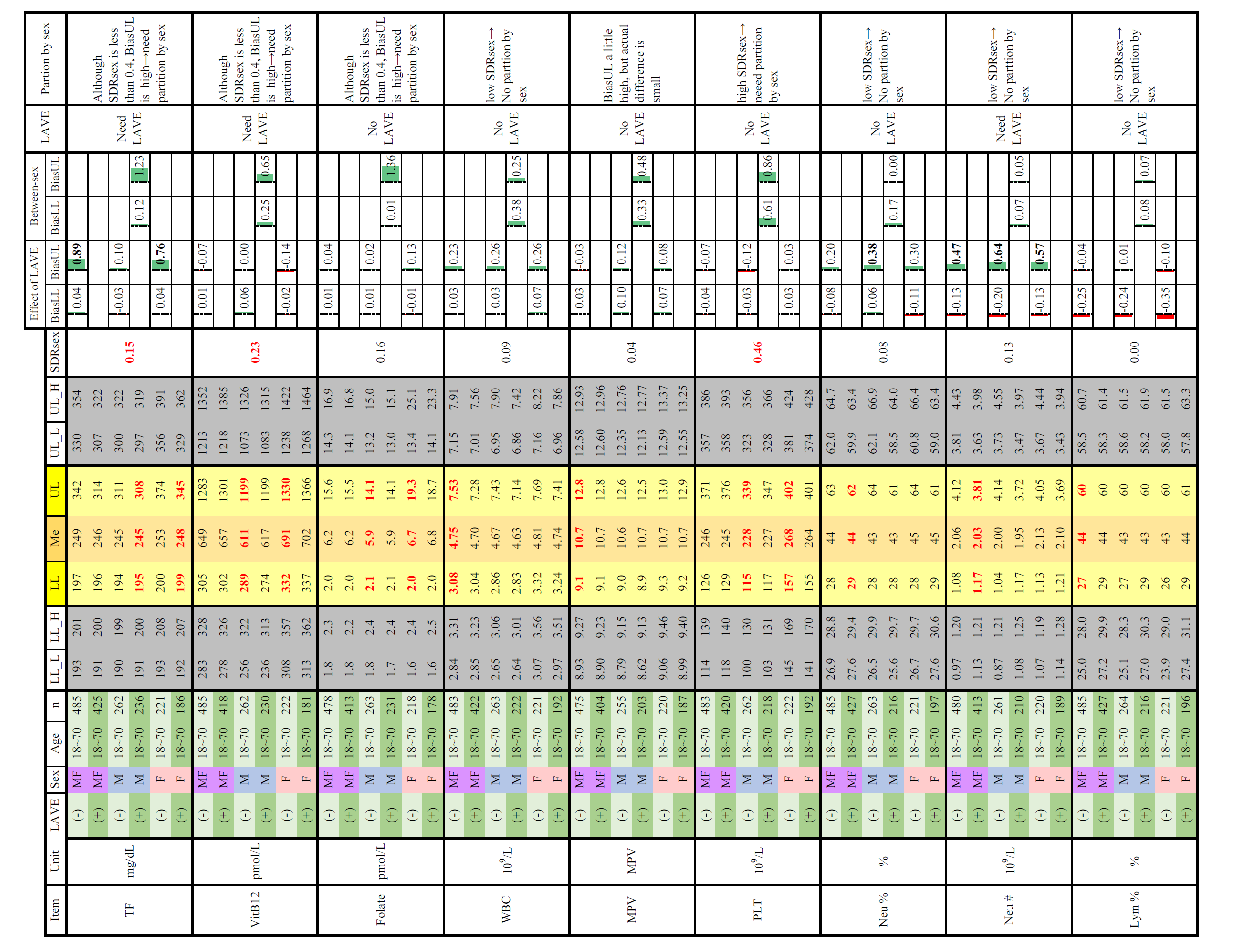
**

**
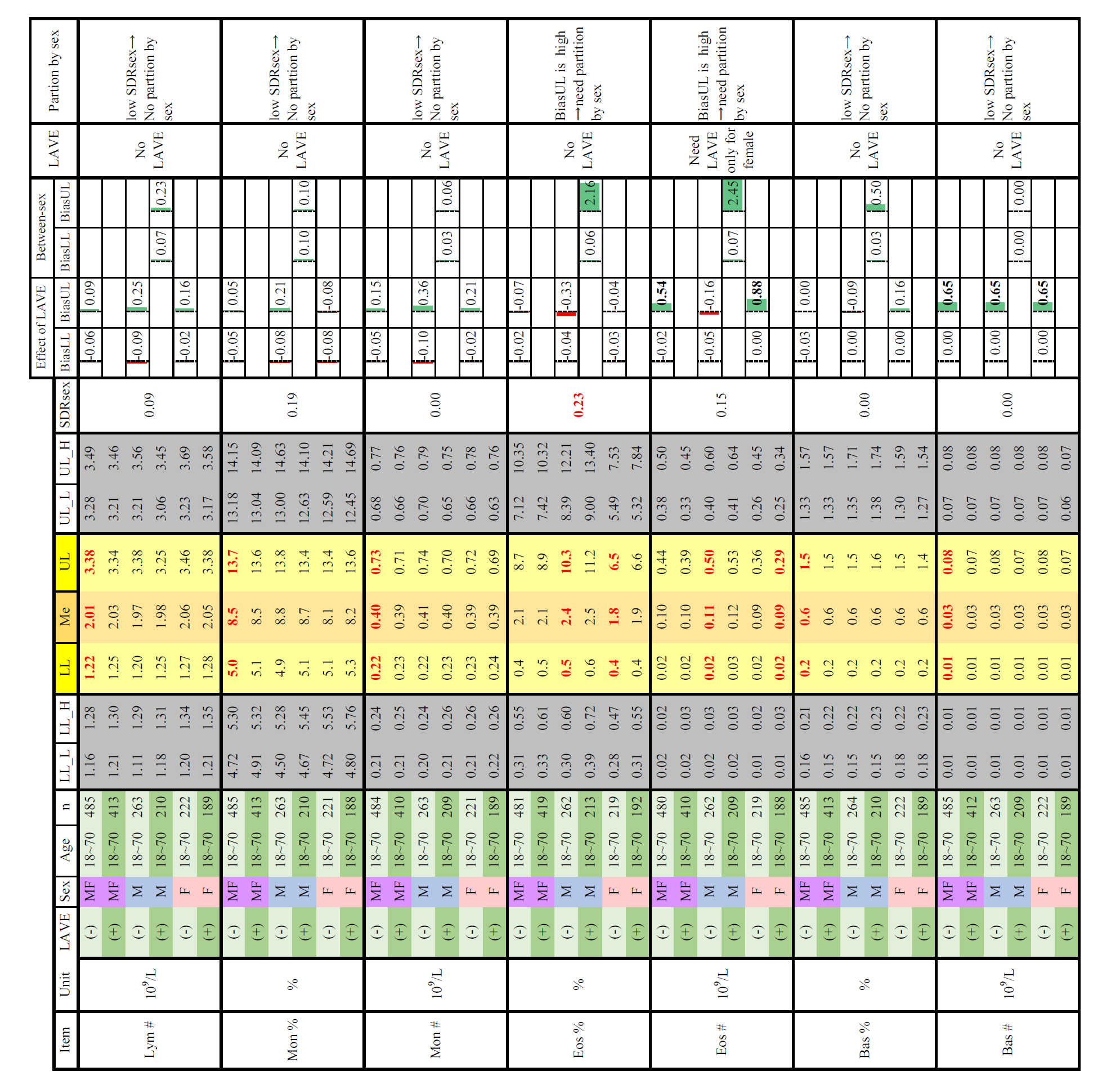
**LL, lower limit of RI; Me, median; UL, upper limit of RI; The RIs we adopted were indicated by values in bold font. The symbol (+) at the LAVE column in the table indicates that RI by the LAVE procedure was adopted based on BiasLL and BiasUL shown in Supplemental Table 2. # indicates absolute counts, whiles % depicts absolute percentage.

**Suppl. Fig 1.**

**
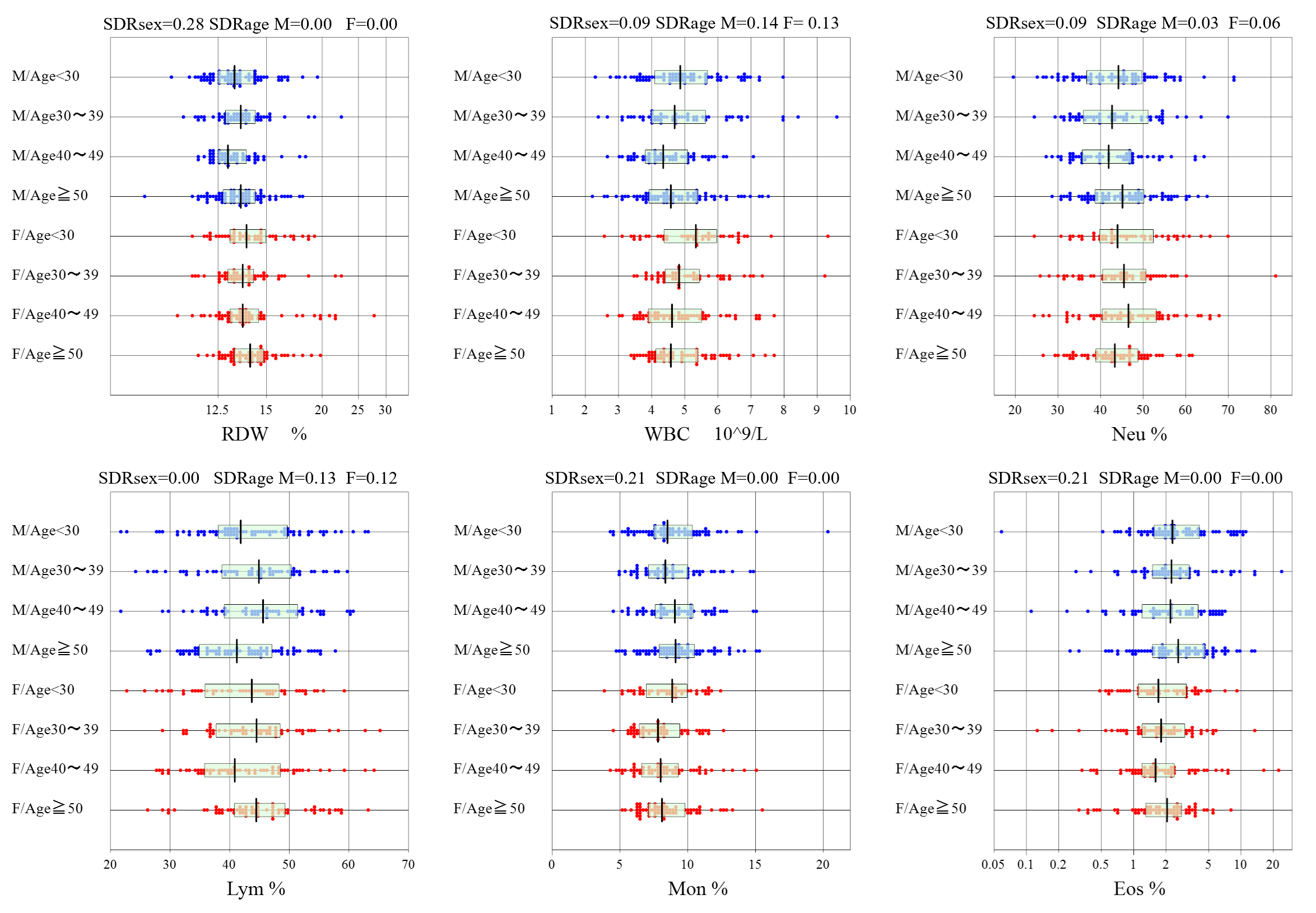

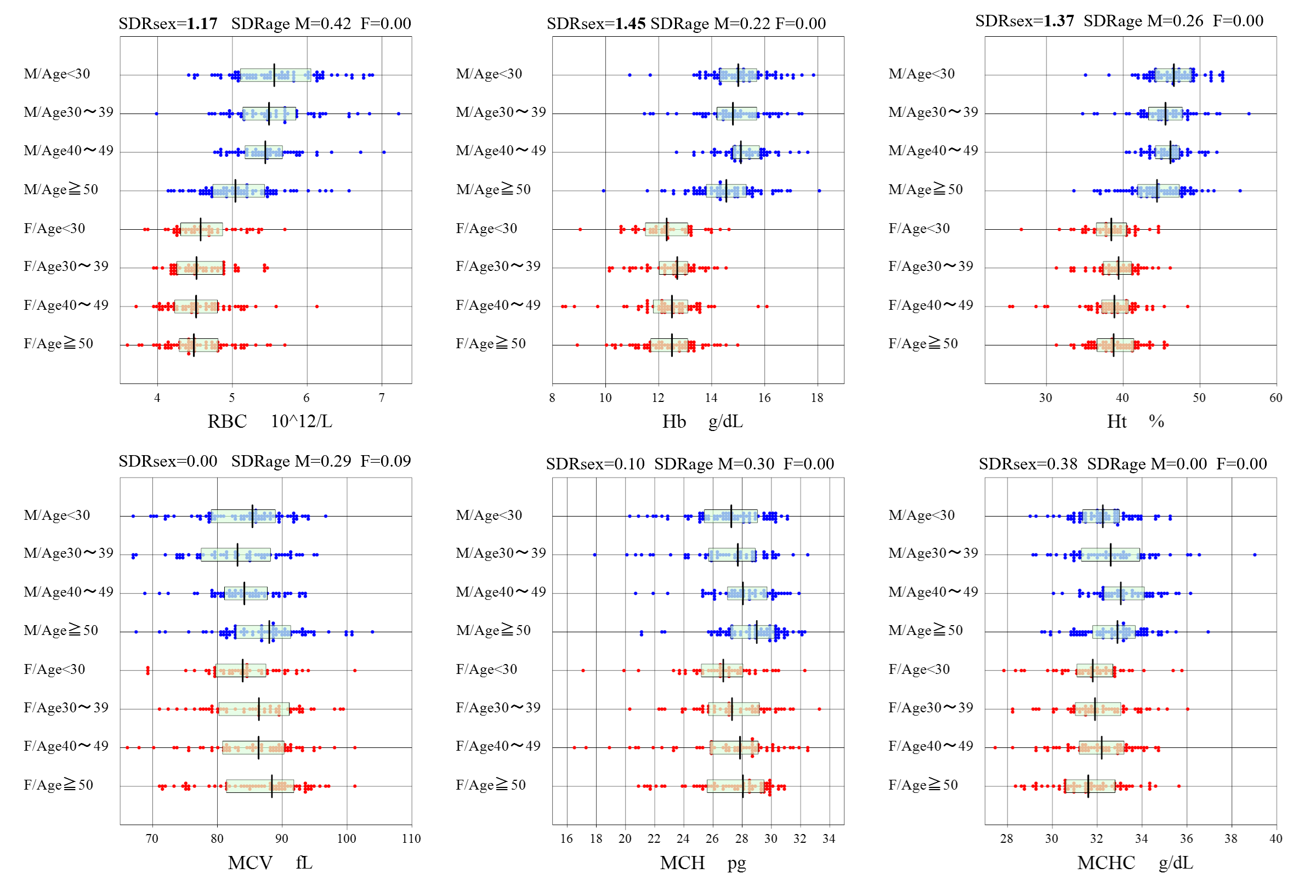
The graphical representation of sex and age-related changes for all the hematological parameters.**

**
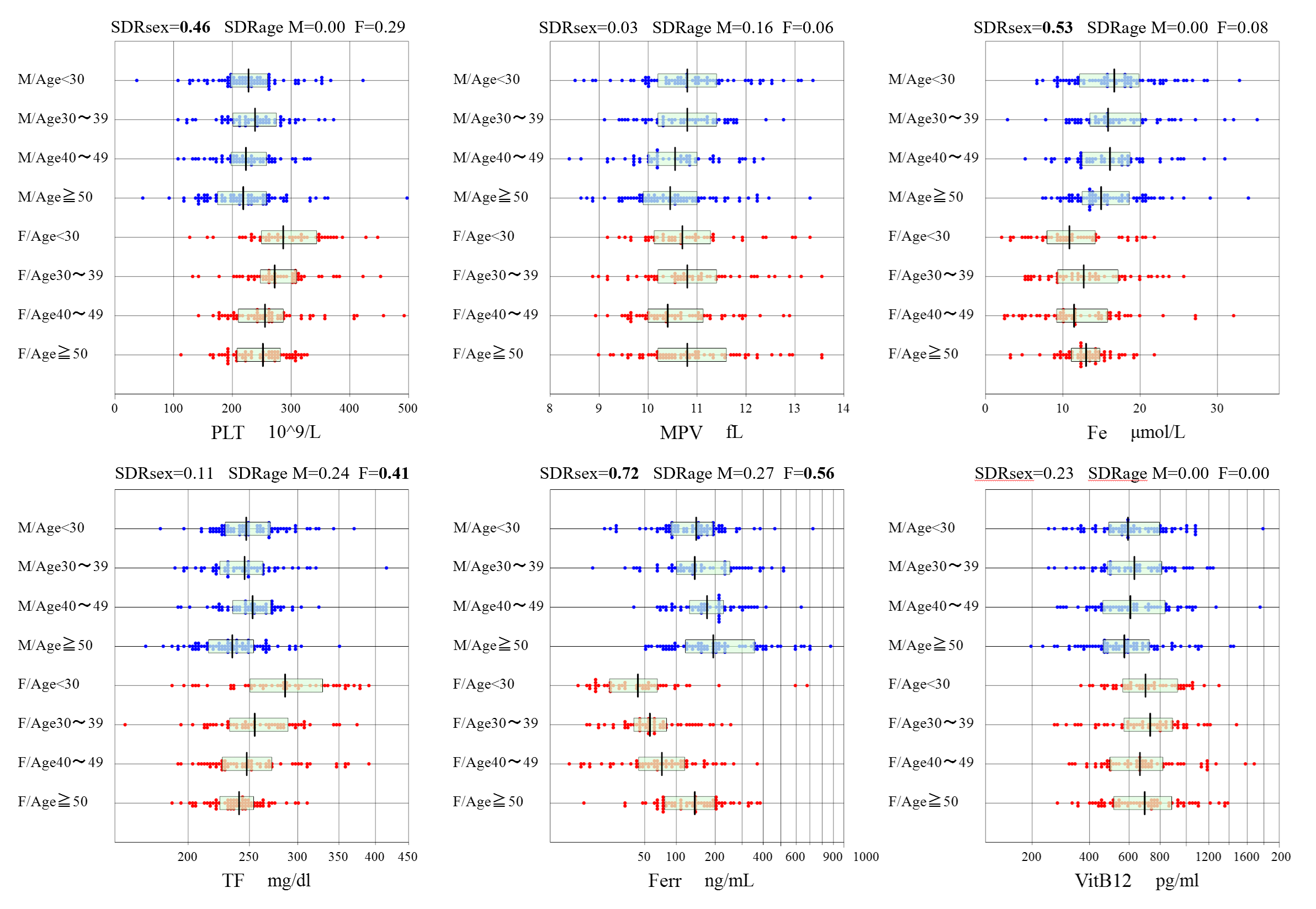

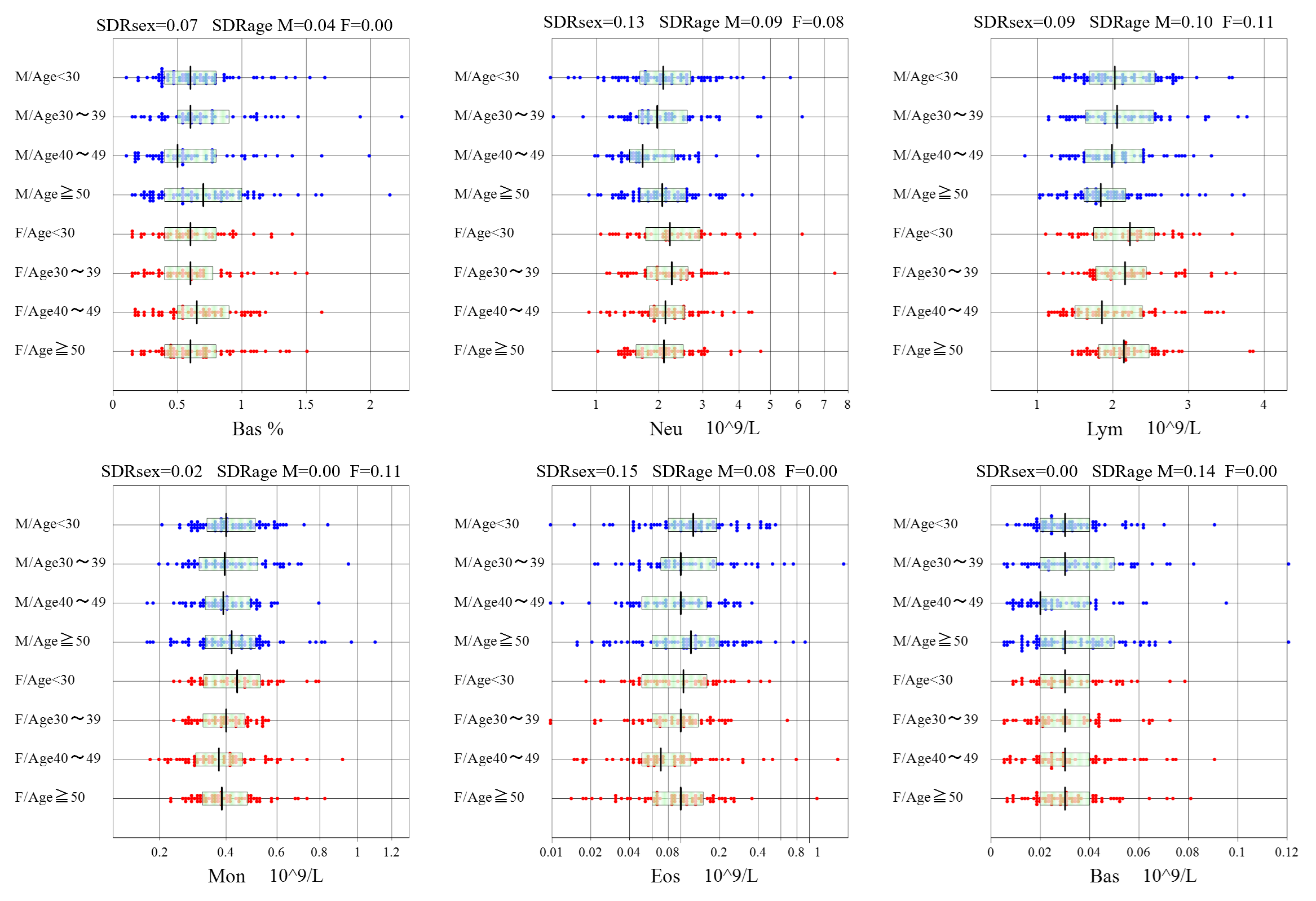
**

**
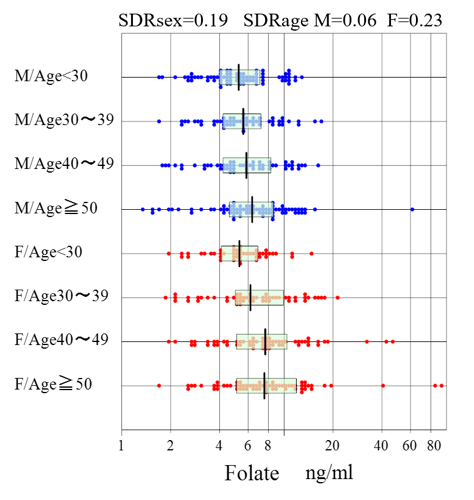
**

All values (RVs) were plotted without any exclusion after stratification by sex and age (in four strata). The blue and red dots represent values of males (M) and females (F), respectively. The box in the center represents central 50% range and the vertical line in its center indicates a median point. The SDRsex was derived by two-level nested ANOVA and SDRage by one-way ANOVA. SDR≥0.4 was marked by bold font

**Suppl. Fig 2 Comparison of hematological RIs among 4 studies conducted in Ghana**

Suppl Fig 2. Graphical representation of hematological reference intervals (RIs) studies conducted in Ghana. The pair of horizontal bars for each study represent RIs for male (M) in blue, and female (F) in red, with the current study depicting the deep blue and red color. The background shades in blue and pink represent RIs of this Ghanaian study for M, and F, respectively. RBC: red blood cells Hb: hemoglobin, Ht: hematocrit, WBC: white blood cell count, Neu#: neutrophil counts, Lym#: lymphocyte counts, Mon#: monocyte counts, Eos#: eosinophil counts and PLT: platelet count.
